# Supplementary material for: Towards sustainable pharmacy practice: Community pharmacists’ experiences with medicine waste, reuse and disposal
Source: PLoS One. 2026 May 15;21(5):e0346163. doi: 10.1371/journal.pone.0346163 (PMC13178855; doi:10.1371/journal.pone.0346163)
Supplement: S1 File — Minimal anonymised data set. (DOCX) [file pone.0346163.s002.docx]

**Interview Responses**

**P 1**

*Interviewer*: The interview is being recorded so if you want me to stop at any moment, just let me know and I'll turn it off.

*Participant*: Right

*Interviewer*: So can you just provide some background on your experience working in pharmacy in Northern Ireland?

*Participant*: I worked in Hospital as clinical pharmacy development for 30 years and I have a community pharmacy of my own for 14 years, so I left the hospital three years ago so now I'm back in the community full time.

*Interviewer*: And what motivated you to pursue this career path?

*Participant*: I experienced it at a level experience and I went to a local pharmacy and it was very impressed what he [pharmacist] did so that that really motivated me. I also like the idea only on businesses at some stage so I think that was it.

*Interviewer*: Perfect. And then just in terms of returning medicines, do you have any current procedures in place for your community pharmacy for return medicines?

*Participant*: We have an open, well, it's blister pack recycling bin, which is always in the dispensary and open and available in the pharmacy. And then when are sealed in the back.

*Interview*: Great. And then do in terms of the environment, do have you taken any measures to minimize wastage in your pharmacy?

*Participant*: I'm trying to figure something out but there is still quite a bit of wastage. What we also find to be honest is we get we could wastage from other pharmacies because where we are people can park at our pharmacy but, I think there's a lot of talk around the food supplements.

*Interviewer*: Perfect. And then do you face any challenges regarding return medicines or wastage in your pharmacy?

*Participant*: No the challenging bit of that goes on for whatever reason the government however the board have set up a collection service which we don't pay for. So no challenges um and obviously with heightened measures, not for waste medicines those take time, but not really no. And the other issues you have to look at for control drugs. When they return you have to make sure they not the mixed with the other returned drugs.

*Interviewer*: Great. And then you mentioned just the guidelines. How do you find now navigating them?

*Participant*: Well measures to be honest you, in my pharmacy, the pharmacist does the disposal because of we want to see what it was return. It's tricky because control drugs are strict only so the pharmacy does all their returns

*Interviewer*: Have you noticed any initiatives local or national that you’ve seen the for the return medicines and disposal?

*Participant*: Sometimes. I haven't seen one in a couple of years now, but it was a turn over medicines promotion scheme, which I think it involves community centres.  So the only thing I would be concerned about is that it needs to be on the supervision of a pharmacist. Well, certainly a pharmacy needs to overview the waste because there is the possibility of diversion. So those kinds of things definitely I think pharmacy is probably best placed those kind of complicated drugs.

*Interviewer*: Definitely. And then in terms of the actual patient, do you ever educate patients about returning the medicines or disposal?

*Participant*: Yes when their medicine has changed. We encourage them to bring the medicines in and we dispose of them properly. A lot of a lot of people just say I'll throw them away but we say no and bring them in and handle medicines properly. Most of our customers are fairly good, but obviously I don't see what goes on at home.

*Interviewer*: In terms of patient attitudes and behaviours, would they'd be pretty good?

*Participant*: The patients would bring in medicine and say we should reuse it. When we inform them we can’t they look very confused, especially when the packaging is still intact. But the rules are the rules.

*Interviewer*: In terms of multidiscipline, do you work with any healthcare professionals relating to these issues?

*Participant*: I used to in the hospital with nurses but substantially less now. Obviously you do work multiprofessional team when you're contacting the GP practices all the time, but yes less so in a community pharmacy.

*Interviewer*: Very good. And just in terms of innovated approaches, can you share any strategies or initiations your pharmacy has implemented to enhance the management returned medicines and disposal.

*Participant*: Recently no, we do see some programs about these medicine returns. But really no.

*Interviewer*: What do you envision and the future developments and improvements in this area within pharmacy?

*Participant*: That's probably something that up for anyone because we don't get that many medicines in to be honest. Yes we really follow whatever national department or board publish. They sort of vote and we get money.

*Interviewer*: Is there anything else you like to add or discuss regarding medicine waste disposable, anything like that?

*Participant*: I suppose for food supplements. I think there's a big food supplement market which results in a lot of waste.

*Interviewer*: Thank so much for your time I truly appreciate it.

**P 2**

Interviewer: Perfect, and just before we start, do I have your consent to record this interview?

Participant: Yes, that’s perfect.

Interviewer: Great. If you ever want the recording to stop, just let me know, and you can withdraw from the interview at any time. Is that okay?

Participant: Yes, that’s fine.

Interviewer: Perfect. To start, can you provide some background on your experience working in community pharmacy in Northern Ireland?

Participant: I've been working in community pharmacy for about 12 years now, mostly in busier pharmacies, but also in smaller, more rural settings. It’s given me a good mix of experience—dealing with the fast-paced environment, regular patients, and the challenges that come with both. No two days are ever the same, which is what keeps it interesting.

Interviewer: And how long have you been involved in pharmacy practice? What motivated you to pursue this career path?

Participant: I’ve been in pharmacy for 12 years now. What motivated me was the opportunity to help people—building relationships with patients, giving advice that makes a real difference, and helping them manage their medications properly. Plus, I enjoy the problem-solving side of things. Every day brings a new challenge.

Interviewer: Perfect. In terms of your current practices, could you describe the procedures in place for managing returned medicines in your pharmacy?

Participant: Controlled drugs have their own process—we have to denature them before disposal, which takes a bit longer. Everything gets collected by a waste disposal company regularly. It’s a fairly routine process, but the volume of returns can be surprising at times.

Interviewer: And what measures are typically taken to minimize medicine wastage in your pharmacy?

Participant: We encourage patients to only order what they actually need. If we notice they are stockpiling or frequently returning medication, we check in with them. We also try to work with GPs to prevent automatic reordering of prescriptions when it’s not necessary. That said, sometimes waste is unavoidable. Once medicines leave the pharmacy, we can’t take them back—even if they’re unopened—which is rather frustrating.

Interviewer: What challenges do you encounter regarding the management of returned medicines and minimizing medicine wastage in your pharmacy?

Participant: The main issue is that some patients don’t return their unused medicines promptly—it just sits at home until they decide to bring it in, or worse, they throw it in the bin. Another challenge is storage. Some days, we receive huge amounts of returned medicines, and there’s only so much space in the pharmacy for waste bins.

Interviewer: Are there any concerns or issues related to the safe disposal of unused or expired medications?

Participant: Yes, mainly environmental concerns and the risk of people improperly disposing of medicines. A lot of people still don’t realize that flushing medicines or throwing them in the bin is harmful. Medications need to be destroyed properly so they don’t cause environmental damage or pose a risk to others.

Interviewer: How do you navigate regulatory requirements and guidelines related to the management and disposal of medicines and expired medications?

Participant: We follow strict protocols to ensure everything is recorded properly and disposed of in line with waste management regulations. It can feel like a lot of admin at times, but it’s necessary to keep things safe and compliant.

Interviewer: Are there any existing policies or initiatives at the local or national level that influence your pharmacy’s practices in this regard?

Participant: There are general awareness campaigns about reducing medicine waste, but beyond that, the policies are quite rigid. Once a medicine is dispensed, it can’t be reused, no matter what. It would be great to see some kind of initiative to safely redistribute unused medicines—perhaps through care homes or hospitals—but at the moment, that’s not an option.

Interviewer: And in terms of patient education, how do you inform patients about the importance of returning unused medications and proper disposal methods?

Participant: Most of the time, when someone collects their medication, we check in with them to see if they actually need everything. We also have posters and leaflets, but to be honest, a quick chat at the counter tends to be the most effective way to get the message across.

Interviewer: Have you observed any changes in patient attitudes or behaviors regarding medicine returns and disposal over time?

Participant: It’s mixed. Some patients are great about bringing back unused medicines, but others still hoard them "just in case." There’s also a bit of reluctance to admit they’ve stopped taking something—it’s like they don’t want to tell their GP they’ve ignored a prescription.

Interviewer: Do you collaborate with other healthcare professionals or organizations to address issues related to medicine returns and disposal?

Participant: Not a large amount, but if we notice a patient regularly returning medicines, we might flag it so that their prescription can be reviewed. In theory, we should all be working together to reduce medicine waste, but communication between pharmacies and GP surgeries isn’t always as good as it could be.

Interviewer: Are there any resources or support systems available to assist community pharmacies in improving their management of returned medicines and minimizing wastage?

Participant: There are guidelines, but beyond that, not really. It’s mostly left up to individual pharmacies to manage things in a way that works best. Some pharmacies have initiatives like prescription synchronization to reduce over-ordering, which can help a bit.

Interviewer: Can you share any innovative strategies or initiatives your pharmacy has implemented to enhance the management of returned medicines and reduce wastage?

Participant: We started trying to do more proactive medicine reviews—checking in with patients before dispensing large repeat prescriptions to make sure they’re still using everything as prescribed.

Interviewer: What do you envision as the potential future developments or improvements in this area within community pharmacy practice?

Participant: I’d love to see some sort of redistribution system for safe, unopened medicines—it seems like a huge opportunity. Also, better integration with GP systems could help reduce unnecessary prescribing in the first place.

Interviewer: And just to close, is there anything else you’d like to add or discuss regarding the management of returned medicines, wastage, and disposal in Northern Ireland pharmacies?

Participant: Pharmacies are doing their best, but we need better awareness from both patients and prescribers. Medicine waste is a big issue, and while we try to manage it at the pharmacy level, there’s only so much we can do without broader policy changes.

Interviewer: Perfect. Thank you so much for your insights and participation in this interview. Would you be open to providing further input or clarification if required in the future?

Participant: Of course.

Interviewer: Thank you so much for your time.

Participant: Thank you.

**P 3**

*Interviewer*: So, can you provide some background on your experience working in community pharmacies in Northern Ireland?

*Participant*: I've been working in pharmacy since I started as a student and worked my way up to managing a pharmacy. It's a busy job, but I enjoy the patient interaction, problem-solving, and keeping everything running smoothly.

*Interviewer*: And how long have you been involved in pharmacy practice? What motivated you to pursue this career path?

*Participant*: I've been in pharmacy for quite a while now, probably longer than I care to admit. I was always interested in healthcare, but I liked the idea of working directly with people in the community rather than in a hospital setting. Pharmacy felt like a good balance between science and patient care, and honestly, no two days are ever the same.

*Interviewer*: In terms of current practices, could you describe the current procedures in place for managing returned medicines in your pharmacy?

*Participant*: Patients bring in used medicines, and we have a process to dispose of them properly. They go into designated waste bins, which are collected by a waste disposal company. We separate out things like controlled drugs, which have to be destroyed under stricter regulations. We also advise patients to return medications rather than bin them at home, especially liquids or sharps.

*Interviewer*: That’s great. What measures are typically taken to minimize medicine wastage in your pharmacy?

*Participant*: We encourage patients to only order what they need. If we notice frequent returns, we check if they might be over-ordering. Unfortunately, there's not much we can do once medications are returned—they have to be destroyed even if they're untouched, which feels wasteful.

*Interviewer*: What challenges do you encounter regarding the management of returned medicines and minimizing wastage?

*Participant*: One challenge is that patients don’t always return medicines promptly, or worse, they dispose of them improperly. Storage is also an issue since large amounts of returns take up space.

*Interviewer*: Are there any concerns related to the safe disposal of unused or expired medications?

*Participant*: Yes, some people still flush tablets down the toilet or throw them in the bin, which isn’t great for the environment. Disposing of controlled drugs is also a process since they have to be denatured before disposal. We try to educate patients, but not everyone listens.

*Interviewer*: In terms of policy and regulations, what are the hygiene and regulatory requirements related to the management and disposal of returned medicines?

*Participant*: We follow guidelines from the Health and Social Care Board (HSCB) and the Medicines and Healthcare products Regulatory Agency (MHRA). There are specific procedures for controlled drugs, hazardous waste, and general medicines, so we ensure everything is recorded and handled correctly by the disposal service.

*Interviewer*: Are there any existing policies or initiatives at the local or national level that influence your pharmacy’s practices in this regard?

*Participant*: Yes, there are government and HSCB initiatives to reduce medicine waste, mostly through patient awareness campaigns. There have been efforts for repeat dispensing to reduce over-ordering, and we sometimes participate in local schemes promoting safe disposal, but ultimately, it depends on patient habits.

*Interviewer*: In terms of patient education, how do you inform patients about the importance of returning unused medications and proper disposal methods?

*Participant*: Mostly through one-to-one conversations at the counter. If someone returns a lot of meds, we ask if they are ordering too much and remind them to only request what they need. We also have posters and sometimes hand out leaflets, but conversations tend to be more effective.

*Interviewer*: Have you observed any changes in patient attitudes or behaviours regarding medicine returns and disposal over time?

*Participant*: There’s been more talk about sustainability and reducing waste, but some people still don’t think about stockpiling meds or just binning them at home. It’s an ongoing effort to get the message across.

*Interviewer*: Do you collaborate with other healthcare organizations to address issues related to medicine returns and disposal?

*Participant*: If a patient is bringing back large amounts of medicine regularly, we might flag it with their GP to adjust their prescription. We also work with local waste disposal services and occasionally participate in health initiatives.

*Interviewer*: Are there any resources or support systems available to assist community pharmacies in improving their management of returned medicines and reducing waste?

*Participant*: We have guidelines from the HSCB, and sometimes we get training or updates from pharmacy organizations like the Pharmaceutical Society of Northern Ireland (PSNI). But a lot of it comes down to experience and common sense—making sure patients understand what they’re ordering and using.

*Interviewer*: Can you share any innovative strategies or initiatives your pharmacy has implemented to enhance the management of returned medicines and reduce wastage?

*Participant*: We’ve tried a few things. One simple but effective approach is having staff double-check repeat prescriptions with patients before dispensing to make sure they actually need everything. Some pharmacies are looking at digital solutions like apps to remind patients to take their meds and reorder only when needed.

*Interviewer*: What do you envision as potential future developments or improvements in this area within pharmacy practice?

*Participant*: Better prescription monitoring between GPs and pharmacies to prevent over-ordering. If there were more safety nets to catch these issues earlier, it would help a lot.

*Interviewer*: Is there anything else you’d like to add regarding the management of returned medicines, wastage, and safe disposal in pharmacies?

*Participant*: Not really, except that while there have been improvements, there’s still a long way to go in changing patient behaviours. Better awareness and technology will definitely help in the future.

*Interviewer*: Perfect. Thank you so much for your insights and participation. Would you be willing to provide further input or clarification if required in the future?

*Participant*: Of course!

*Interviewer*: Thank you so much.

**P 4**

*Interviewer*: Just to let you know when we start this entry it will be recorded. Do I have your consent to do that?

*Participant*: Yes, you do.

*Interviewer*: And any time throughout the recording, you can tell me and I'll turn it off and you can be withdrawn from the participation. Is that okay?

*Participant*: Yes thank you.

Interviewer: So just to start, can you please provide me on background on your experience working on community pharmacy?

*Participant*: Well, I've been working in a community pharmacy say for about six years now, maybe a way bit over. I started off as a locum after qualifying. but I wanted a more permanent role and I stepped into some management. I've worked in a a small independent pharmacy and a larger one, so seen quite a range of approaches to pharmacy practice in my time.

*Interviewer*: Very good. And how long have you been involved in pharmacy practice and what motivated you to pursue this career path?

*Participant*: Well, probably since at school I always liked science um and pharmacy felt like a good mix of problem solving and health care. um and I like I like the idea of working with people, you know, rather than being stuck in an office or a lab or anything like that. So um and I know far our community pharmacies play a big role in people's lives and and, you know, they don't get to see their doctor often, so that keeps me going and keeps me interested in dealing with those sort of people.

*Interviewer*: Great. So just in terms of your practice practices, could you describe the current procedures in place for managing return medicines in your pharmacy?

*Participant*: Well, we have dedicated bins in our pharmacy. So when people bring back their expired medicines or unused medicines, we can use the bins for safe disposable. . And there's lots of there's a few different categories, you know, hazardous, controlled, drugs, general meds, and we just ensure that they go into the right bin. And then there's a licensed waste company um that'll come and collect everything for us and there's a strict process uh that they have to adhere to um before disposal.

*Interviewer*: Perfect. And then just in terms of the environment, what measures are typically taken to minimize medicine wastage in your pharmacy?

*Participant*: Well, I feel that patient education is very important, so a lot of it is down to careful dispensing and making the patient aware. We always check the people actually need their repeat prescriptions before dispensing them, especially things like creams and healers things that tend to pile up at home that probably they don't get used a lot um we try to work with the GP's to work to reduce overprescribing, but that's obviously can be a bit tricky um you don't always be able to see uh the patient's medication history.

*Interviewer*: Perfect. And then what challenges do you encounter regarding the management of return medicines and minimizing medicine wastage in the pharmacy?

*Participant*: Well, I think the main big one is the fact that they can't be reused once they are dispensed, even if the packs are saved, so this is a big issue. When people bring back a large amount of unused medications, it just seems like a waste. You know, when we're a busy pharmacy and we have these waste bins, they take up a lot of storage space. um so we have to stay on top of collections and of course some patients just don't think about the waste of medicine. um so it's just ongoing trying to educate them um but it is a big issue.

*Interviewer*: And are there any uh concerns or issues related to the safe disposable of unused or expired medications?

*Participant*: Well, what we find is a lot of patients will still throw tablets into the bin or flush them down the toilet, which obviously can be harmful to the environment. So, disposing of medication properly is a big problem, especially we have to be really careful with control drugs and certain hazardous medicines, as they require specific handling, we obviously have to ensure that they won't end up in the wrong hands.

*Interviewer*: In terms of policy and regulatory, how you do navigate regulatory requirements and guidelines related to the management and disposal of return medicines and expired medications?

*Participant*: Well, there are very clear rules on how to store and dispose at medicine safely, and we follow the guidelines from two organisations, the first one, the health and social care board um and then the pharmaceutical society of Northern Ireland. They have clear rules on how to store and dispose and they give us regular updates so just to make sure that we are still doing the things the way it's supposed to be done. Ah there can be a lot of paperwork involved sometimes, but it's all it's all for patient safety, which is obviously priority.

*Interviewer*: Are there any existing policies or initiatives at the local or national level that influence your pharmacies practices in this regard?

*Participant*: Well, we know that there have been some campaigns to raise awareness about medicine waste, and we do see efforts people trying government departments trying to improve, repeat prescription processes to cut down on unnecessary dispensing, but really um the waste will always be inevitable because in terms of disposal, you know it's quite rigid, and once medicines leave the pharmacy safe, they cannot be reused so really there's always going to be some sort of waste.

*Interviewer*: And then in terms of patient education and engagement, how do you educate patients about the importance of returning on used medications on proper disposal methods?

*Participant*: Well, we try and make it easy for the patient. We tell them, you know, if you've got medications that you don't need, please bring them back to us rather than throwing them away. Try and make it simple for them. We remind them at the counter. We have posters up and if we are having a consultation, we will explain it to them. But honestly, you know, people respond better to a quick chat so we talk to them as much as we can and when we see them at the counter.

*Interviewer*: And how since working have you observed any changes in patient attitudes or behaviours regarding medicine returns and disposal over time?

*Participant*: Well, some patients are definitely becoming more aware, especially about the environmental impact, but we do feel we still have a long way to go. I mean, most people, they just come in and they don't realize that they're hoarding medication at home until they have a clear out by then it's probably too late to do anything about it and again, you know, they may not know even what to do with it

*Interviewer*: And then in terms of collaboration, do you collaborate with other healthcare professionals or organizations to address issues related to medicine returns on disposal?

*Participant*: Well, um maybe with the GP and sometimes maybe a district nurse um if we notice say a patient continually bringing them to see if their prescription needs reviewing. But sometimes um we do get involved in local initiatives about reducing the waste, but there's definitely a lot more room for more collaboration.

*Interviewer*: And are there any resources or support systems available to assist community pharmacies and improvement improving their management over term medicines and wastage?

*Participant*: Well, we do have things like training sessions, and of course there are official guidelines as well, but it's mostly down to individual pharmacies to implement their best guides and practices. But it would be great to see a more structured support or incentives for pharmacies to take a more active role in medicine waste.

*Interviewer*: And then just in terms of thinking of the future, can you share any initiative strategies or in shows your pharmacy is implemented to enhance the management over term medicines and just wastage?

*Participant* : Well, it might be just seeing just a simple step, but one thing we do is to check the patient with the patient before dispensing their repeats, but it can make a big difference in reducing unnecessary prescriptions. I heard of some pharmacies that are trailing digital reminders to help patients manage their medication better, which could be good in the long run.

*Interviewer*: And what do you envision as a potential future developments or improvements in this area within community pharmacy practice?

*Participant*: Well, I think we could make a better improvement with better coordination between pharmacies and GPs. This would be such a big step forward. If we had, say, real time prescription tracking, we could catch issues like over ordering earlier. Also, if there was a safe way to redistribute unopened return medicines rather than destroying them, that would be a big game changer. But that could be tricky. I imagine the regulatory side of that would be very complicated.

*Interviewer*: Then just to close, is there anything else you would like to add or discuss regarding the management of term medicines, wastage and disposal in community pharmacies?

*Participant*: Well, as we said earlier, it's still a big problem. and it's an ongoing challenge continually. We do our best at the pharmacy to minimise a waste, but a lot of it does come down to the patient behaviour and prescribing practices of the GP. overall, I think we just need more awareness and maybe some better systems in place, but it could definitely help in the future.

*Interviewer*: Fantastic thank you so much for your insight and participation in this interview. Would you be willing to provide further input or clarification if needed in the future?

*Participant*: Absolutely. I'm happy to help. Let me know if there's anything else you need.

*Interviewer*: Perfect, thank you so much for your time.

**P 5**

**Interviewer:** So to start, can you just provide some background on your experience working in community pharmacies in Northern Ireland?

**Participant:** Yeah, sure. I’ve been working in a community pharmacy here in Northern Ireland for about 4 years now. It’s been a great experience. You get to know your regular patients, help them manage their medicines, and sometimes even just have a chat, which is nice. It can be a fast-paced environment, but I like the mix of routine and the unexpected.

**Interviewer:** Thank you. And how long have you been involved in pharmacy practice, and what motivated you to pursue this career?

**Participant:** I’ve been in pharmacy since my studies, but even during my studies, I was working part-time in a pharmacy. I was always interested in healthcare, but I liked the idea of working in the community rather than a hospital setting. Plus, there’s a good mix of science and patient care, which keeps it interesting.

**Interviewer:** Perfect. And could you describe the current procedures in place for managing returned medicines in your community pharmacy?

**Participant:** Yeah, so when patients bring medicines back, we have specific bins for disposal—separate for general medicines, controlled drugs, and hazardous waste. Everything gets logged and then collected by the waste disposal company. Controlled drugs have stricter rules—we have to denature them before disposal. So, yeah, it is quite a lot.

**Interviewer:** And what measures are typically taken to minimize medicine wastage in your pharmacy?

**Participant:** So we try to encourage patients to only order what they actually need. That said, waste is sometimes unavoidable. Once the medicine leaves the pharmacy, we can’t take it back, even if it’s unopened, which is unfortunate.

**Interviewer:** And in terms of challenges, do you currently face any regarding the management of returned medicines and minimizing medicine wastage in your pharmacy?

**Participant:** We get a huge amount of medicines, and there’s only so much space in the pharmacy for bins. Another issue is that some patients don’t return medicines at all. They just sit at home or, worse, get thrown in the bin.

**Interviewer:** Are there any concerns or issues related to the safe disposal of unused or expired medications?

**Participant:** Yeah, definitely. A lot of people still don’t realize that flushing medicines down the toilet or throwing them in the bin is a bad idea. Some medicines can be harmful to the environment, and controlled drugs, in particular, need to be disposed of properly and legally.

**Interviewer:** How do you navigate regulatory requirements and guidelines related to the management and disposal of returned medicines and expired medications?

**Participant:** Yeah, so we follow the guidelines set by the government and the PSNI. There’s a lot of documentation to ensure everything is recorded properly, but it’s necessary to keep things safe and compliant. But I must say it can be quite tricky navigating the guidelines sometimes. I feel like some information overlaps, and some information I can’t find.

**Interviewer:** Are there any existing policies or initiatives at the local or national level that influence your pharmacy’s practice in this regard?

**Participant:** The government runs campaigns to raise awareness about medicine waste and efforts to improve repeat prescription processes to cut down on unnecessary dispensing, but realistically, waste will always be an issue because once medicines are dispensed, they can’t be reused, even if they’re unopened.

**Interviewer:** And in terms of the patient, how do you educate patients about the importance of returning unused medications and proper disposal methods?

**Participant:** Yeah, mostly through direct conversations with the patient. If someone returns a lot of medicines, we’ll ask if they’re over-ordering or even if they need the actual medication. We try to remind them to only request what they need. We also have posters and sometimes hand out leaflets, but honestly, you don’t get a lot of information to give out to patients regarding medicine wastage.

**Interviewer:** Have you observed any changes in patient attitudes or behaviors regarding medicine returns and disposal over time?

**Participant:** There’s definitely more awareness now, especially around the environmental impact, but there’s still a long way to go. Some people still hoard meds at home without realizing how much builds up over time. I don’t think they correlate it to the environmental impact just yet. An example I can think of is the CFC-free inhaler. People were still quite confused about why their inhalers were being switched, but I guess that’s probably just due to not educating the patient.

**Interviewer:** And in terms of collaboration, do you collaborate with other healthcare professionals or organizations to address issues related to medicine return and disposal?

**Participant:** Occasionally, I guess so. If we notice a patient is bringing back a lot of unused medicines regularly, we might flag it to their GP to review the prescription. But in general, there isn’t much structured collaboration on this issue.

**Interviewer:** Are there any resources or support systems available to assist community pharmacies in improving their management of returned medicines and medicine wastage?

**Participant:** There are official guidelines, but honestly, it’s mostly left up to individual pharmacies to implement the best practices. You just try to do your best and use your own experience.

**Interviewer:** And can you share any innovative strategies or initiatives your pharmacy has implemented to enhance the management of returned medicines and reduce medicine wastage?

**Participant:** A simple thing is checking in with patients before dispensing the repeat to make sure they actually need everything, but most of the time, we just let the patient manage their own medicines independently if they are able to. Most are sensible, but at the same time, some aren’t even aware that you can return the medicines back to the chemist.

**Interviewer:** And what do you envision as a potential future development or improvement in this area within pharmacy practice?

**Participant:** I think if pharmacists had access to the entire patient records, it would be a huge step forward. If we had real-time prescription tracking, we could catch over-ordering issues earlier. Also, if there was a possibility of dispensing returned unopened medicines, it would reduce waste, but I don’t know about the safety and legality of that.

**Interviewer:** And then, just to wrap up, is there anything else you’d like to add or discuss regarding the management of returned medicines, wastage, and disposal in community pharmacies?

**Participant:** I think there are definitely resources to manage returned medications, but I think there is room for additional steps to be made to make a bigger impact on the environment. But I know that is probably very much in the future.

**Interviewer:** And thank you so much for your insight and participation in this interview. Will you be willing to provide further input or clarification if required in the future?

**Participant:** Yeah, sure.

**Interviewer:** Thank you so much for your time.

**P 6**

**Interviewer:** So just to start, can you provide some background on your experience working in community pharmacies in Northern Ireland?

**Participant:** Absolutely. I've been in pharmacy for decades now—over 35 years, actually. I started in a small independent pharmacy back in the day, worked my way through different roles, and eventually took on a management position. Now I run a busy urban pharmacy, which comes with its own set of challenges, but I wouldn’t trade it for anything. The industry has changed a lot over the years, and you have to keep up, but at the heart of it, it’s still about patient care.

**Interviewer:** And how long have you been involved in pharmacy practice, and what motivated you to pursue this career path?

**Participant:** It wasn’t exactly a childhood dream, if I’m honest, but I was always interested in healthcare, and pharmacy seemed like a good idea. Plus, I liked the idea of being in a role where you could build long-term relationships with patients. As the years went on, I found I enjoyed the management side of things too, running a pharmacy, looking after a team, making sure everything runs smoothly.

**Interviewer:** And can you describe the current procedures in place for managing returned medicines in your pharmacy?

**Participant:** When medicines are brought back, we have to handle them carefully. There’s a strict process in place, especially for controlled drugs that need to be documented and disposed of properly; otherwise, we could run into legal issues. Everything else gets sorted into different categories, and we have a disposal company that comes to take it all away. But honestly, it’s not as streamlined as it could be. Some days we get so much back that we’re practically swimming in returns, and finding the space to store them until collection can be a real headache.

**Interviewer:** And what measures are typically taken to minimize medicine wastage in your pharmacy?

**Participant:** We do our best to keep track of what patients are ordering. If we notice someone getting a large number of repeats and then returning them, we’ll have a chat with them or their GP to see if they actually need everything. But it’s tough—once a prescription is written and collected, there’s not much we can do if it turns out they didn’t need it. Some people also stockpile meds at home because they don’t want to bother their doctor, and then months later, they bring in bags of unused tablets.

**Interviewer:** What challenges do you encounter regarding the management of returned medicines and minimizing medicine wastage in your pharmacy?

**Participant:** One of the biggest issues is just sheer volume. Some days we get a handful of returns, and other days it’s like half the city has decided to clear out their medicine cabinets at once. We don’t have unlimited space, and when those bins start filling up, it becomes a logistical nightmare. Another issue is patients thinking we can reuse returned meds. I totally get why it seems like a waste to throw away unopened packs, but unfortunately, the rules are strict.

**Interviewer:** Are there any concerns or issues related to the safe disposal of unused or expired medications?

**Participant:** Absolutely. A lot of people still don’t realize how harmful it is to just toss medicine in the bin or flush it down the toilet. We’ve had patients tell us they’ve done it without thinking, and it’s worrying. Controlled drugs are another big concern. We have to be extra careful they’re disposed of properly because the last thing we want is for them to be misused.

**Interviewer:** And how do you navigate regulatory requirements and guidelines related to the management and disposal of returned medicines and expired medications?

**Participant:** If I’m being completely honest, the regulations are a pain. They’re necessary, of course, but they’re not always practical. The amount of paperwork involved in controlled drug disposal is ridiculous, and it feels like the rules keep changing, which makes it hard to keep up.

**Interviewer:** Are there any existing policies or initiatives at the local or national level that influence your pharmacy’s practice in this regard?

**Participant:** There have been some attempts at awareness campaigns to reduce waste, and we get the odd update from regulatory bodies, but in terms of actual support—not much. The rules around medicine returns are rigid, so there’s not much flexibility.

**Interviewer:** And in terms of patient education, how do you educate patients about the importance of returning unused medications and proper disposal methods?

**Participant:** We try to explain it at the counter when people collect their prescriptions, but it’s a lot of information to take in. We also have posters up and leaflets available, but let’s be honest—most people don’t bother reading them.

**Interviewer:** Have you observed any changes in patient attitudes or behaviors regarding medicine returns and disposal over time?

**Participant:** There’s been a slight improvement. I’d say more people seem to be aware that they should bring back unused meds instead of binning them, but there are still plenty who hoard their medicines at home—either because they’re worried they might need them later or just forget to clear them out.

**Interviewer:** And in terms of collaboration, do you collaborate with other healthcare professionals or organizations to address issues related to medicine returns and disposal?

**Participant:** Not as much as I’d like. We sometimes flag issues to GPs if we notice a pattern of excessive returns, but there isn’t really a structured system for it. Ideally, there’d be better coordination between pharmacies and GP practices to prevent overprescribing in the first place, but that’s easier said than done.

**Interviewer:** Are there any resources or support systems available to assist community pharmacies in improving their management of returned medicines and medicine wastage?

**Participant:** There are some guidelines, but beyond that, it’s mostly left to pharmacies to handle things their own way. It would be great if there was funding to help pharmacies manage medicine returns better or even a centralized system to deal with waste more efficiently.

**Interviewer:** And can you share any innovative strategies or initiatives your pharmacy has implemented to enhance the management of returned medicines and reduce medicine wastage?

**Participant:** We’ve started doing a bit more proactive prescription monitoring—just asking patients before dispensing large amounts of repeat meds if they actually need all of them. It’s a small step, but it helps reduce unnecessary dispensing. But there’s still a lot of room for improvement.

**Interviewer:** And what do you envision as a potential future development or improvement in this area within pharmacy practice?

**Participant:** I think better integration between pharmacies and GP systems would make a huge difference. And if there was a way to safely redistribute unopened medicines instead of destroying them, that would be pretty good.

**Interviewer:** And just to close, is there anything else you’d like to add or discuss regarding the management of returned medicines, wastage, and disposal in community pharmacies?

**Participant:** Just that pharmacies are trying their best, but we need more support from the system. More awareness and better policies will go a long way in tackling this issue properly.

**Interviewer:** Thank you so much for participating in this interview. Would you be willing to provide further input if needed in the future?

**Participant:** Of course—happy to help anytime.

**Interviewer:** Thank you so much.

**P 7**

**Interviewer:** So just to start, can you provide some background on your experience working in community pharmacies in Northern Ireland?

**Participant:** Yeah, so I’ve been working in community pharmacy for about six years now. It’s a busy place—being in an urban area, we have a lot of different patient needs, and it can get a bit hectic, to be honest, but I enjoy it.

**Interviewer:** And how long have you been involved in pharmacy practice, and what motivated you to pursue this career path?

**Participant:** I’ve always been interested in healthcare, but I wanted to do something where I could interact with people day to day rather than being stuck in a lab or hospital ward. I think I wanted to give back to my community, and pharmacy felt like the best way to do it.

**Interviewer:** And so can you describe the current procedures in place for managing returned medicines in your community pharmacy?

**Participant:** Yeah, so patients bring in unused or expired meds, and we put them in our waste bins. The waste company comes to collect it, and controlled drugs need to be denatured before disposal. There’s quite a bit of paperwork involved, and honestly, I sometimes feel like I spend half my day just filling out forms.

**Interviewer:** And what measures are typically taken to minimize medicine wastage in your pharmacy?

**Participant:** Yeah, we always try to check if people actually need all their repeat prescriptions before dispensing, but it’s not always easy. Some patients just request everything automatically. We also encourage people to only order what they’ll use, and if we see regular returns, we might mention it to their GP. But honestly, once a medicine is dispensed, it’s out of our hands, and a lot of it ends up being wasted.

**Interviewer:** And then what challenges do you encounter regarding the management of returned medicines and minimizing medicine wastage in your pharmacy?

**Participant:** One big issue is that people don’t always bring medicines back promptly. They hold on to them for months, and by the time they return them, they’re way past their expiry date. Another problem is if a patient brings back a mix of drugs—we have to go through everything and make sure there are no needles, controlled drugs, or anything like that, which can take a while to do.

**Interviewer:** And are there any concerns or issues related to the safe disposal of unused or expired medications?

**Participant:** Definitely. A lot of people still don’t realize that chucking meds in the bin or flushing them down the toilet is a big no-no. It’s worrying because some medicines can be harmful if they get into the water supply. We try to educate people, but not everyone listens.

**Interviewer:** And how do you navigate regulatory requirements and guidelines related to the management and disposal of returned medicines and expired medicines?

**Participant:** If I’m being honest, I find the guidelines pretty confusing at times. There’s a lot of paperwork involved, and the rules around controlled drugs, in particular, can be quite complicated. The guidelines keep changing, and it feels like there’s always something new to keep on top of. Sometimes I have to double-check things just to make sure I’m doing it right.

**Interviewer:** And are there any existing policies or initiatives at the local or national level that influence your pharmacy’s practices in this regard?

**Participant:** So yeah, there have been a few awareness campaigns about reducing medicine waste, but beyond that, the policies are quite strict. Once a medicine has been dispensed, it can’t be reused, no matter what. It would be great if there was some system to safely redistribute unopened meds instead of destroying them, but right now, that’s not an option.

**Interviewer:** And in terms of patient engagement, how do you educate patients about the importance of returning unused medicines and proper disposal methods?

**Participant:** We try to mention it when people collect their prescriptions—just a quick reminder that if they don’t need something anymore, they should bring it back. We also have posters up in the pharmacy, but let’s be honest—most people don’t stop to read them. A lot of it comes down to those little one-on-one conversations at the counter.

**Interviewer:** And have you observed any changes in patient attitudes or behaviors regarding medicine returns and disposal over time?

**Participant:** A bit, yeah. More people are aware that they shouldn’t just throw meds away, but there are still a lot of patients who hoard them just in case. And when we tell them we can’t reuse their unopened packs, they’re usually surprised or frustrated. I think they believe they are helping us, but there’s no way we can reuse those medications.

**Interviewer:** OK, and then in terms of collaboration, do you collaborate with other healthcare professionals or organizations to address issues related to medicine returns?

**Participant:** Not as much as I think we should. We sometimes flag things to GPs if a patient is constantly returning meds, but there’s not much direct collaboration. It would be helpful if there were better communication between pharmacies and GP surgeries to prevent overprescribing in the first place, but I guess we are all under pressure these days.

**Interviewer:** And are there any resources or support systems available to assist community pharmacies in improving their management of returned medicines and medicine wastage?

**Participant:** We get guidelines, but that’s about it. Most of it is left up to individual pharmacies to figure out the best way to handle things. Some kind of structured support or even funding to help pharmacies manage returns better would make a big difference.

**Interviewer:** And can you share any innovative strategies or initiatives your pharmacy has implemented to enhance the management of returned medicines and reduce medicine wastage?

**Participant:** Well, we’ve started being more proactive in checking with patients before dispensing repeat prescriptions, just asking if they actually need everything. But honestly, it’s not something you get trained for, and it can be quite limited in what we can do.

**Interviewer:** And what do you envision as the potential future development or improvement in this area within pharmacy practice?

**Participant:** If there was a legal way to redistribute safe, unopened meds rather than binning them, that would be amazing, but I imagine the regulations around that would be tough to change.

**Interviewer:** And then just to close, is there anything else you’d like to add or discuss regarding the management of returned medicines, wastage, and disposal in community pharmacies?

**Participant:** Well, you know, we can only do so much at the pharmacy level—there need to be bigger policy changes to tackle waste properly. But something does have to change.

**Interviewer:** Thank you so much for participating in this interview. Would you be willing to provide further input or clarification if required in the future?

**Participant:** Yeah, sure, no problem.

**Interviewer:** Lovely, thank you so much for your time.

**Participant:** Thank you.

**P 8**

**Interviewer:** So, to start, can you provide some background on your experience working in community pharmacies in Northern Ireland?

**Pharmacist:** Absolutely. I've been working as a community pharmacist in Northern Ireland for over 10 years now. I started my career after completing my Master's in Pharmacy. Over the years, I've worked in both urban and rural settings, which has given me a well-rounded perspective on the unique challenges and opportunities in community pharmacy practice here.

**Interviewer:** And how long have you been involved in pharmacy practice, and what motivated you to pursue this career path?

**Pharmacist:** I've always been passionate about healthcare and helping people. Pharmacy, in particular, appealed to me because it combines science with direct patient care. Being a community pharmacist allows me to build relationships with patients, provide advice, and play a key role in improving public health outcomes. It's incredibly rewarding to know that my work makes a tangible difference in people's lives.

**Interviewer:** In terms of your current practices, can you describe the current procedures in place for managing returned medicines in your community pharmacy?

**Pharmacist:** Certainly. In our pharmacy, we have a dedicated medicines return bin where patients can drop off unused or expired medications. We follow strict guidelines to ensure these medicines are stored securely until they're collected by a licensed waste disposal company. Unfortunately, we're not permitted to reuse any returned medications, even if they're unopened, due to safety and regulatory concerns.

**Interviewer:** And what measures are typically taken to minimize wastage in your pharmacy?

**Pharmacist:** We take a proactive approach to minimize wastage. For example, we encourage patients to only order the medications they need and to check their supplies at home before requesting repeats. We also advise patients to regularly review their medications for expiry dates. Additionally, we work closely with prescribers to ensure prescriptions are appropriate and tailored to the patient's needs, which helps reduce overprescribing.

**Interviewer:** In terms of challenges, what challenges do you encounter regarding the management of returned medicines and minimizing medicine wastage in your pharmacy?

**Pharmacist:** One of the biggest challenges is the lack of clear, consistent guidelines on safe medicine disposal in Northern Ireland. While we have a system in place, it can be confusing for both patients and pharmacy staff. Another issue is patient awareness—many people aren't aware that they can return unused medicines to the pharmacy, which leads to improper disposal at home.

**Interviewer:** Are there any concerns or issues related to the safe disposal of unused or expired medications?

**Pharmacist:** Yes, improper disposal of medications can have serious consequences for the environment and public health. For example, flushing medications down the toilet or throwing them in the bin can contaminate water supplies or end up in landfills. This not only harms the environment but also increases healthcare costs in the long run. That's why we're so committed to promoting safe disposal practices.

**Interviewer:** And then in terms of policy, how do you navigate regulatory requirements and guidelines related to the management and disposal of returned and expired medications?

**Pharmacist:** We adhere to the guidelines set by the Department of Health and the Pharmaceutical Society of Northern Ireland. However, as I mentioned earlier, the guidelines can be a bit unclear at times. To ensure compliance, we work closely with our waste disposal partners and regularly review our procedures. We also stay updated on any changes in legislation or best practices.

**Interviewer:** Are there any existing policies or initiatives at the local or national level that influence your pharmacy's practices in this regard?

**Pharmacist:** Yes, there are government initiatives aimed at promoting safe medicine disposal, and we fully support these efforts. For example, the Take-Back scheme encourages patients to return unused medicines to pharmacies. While these initiatives are a step in the right direction, I believe more could be done to standardize and enforce these practices across all community pharmacies.

**Interviewer:** Perfect. And then just in terms of patient engagement, how do you educate patients about the importance of returning unused medications and proper disposal methods?

**Pharmacist:** Education is a big part of our role. We use posters, leaflets, and one-on-one conversations to inform patients about the importance of returning unused medicines. We also explain the environmental and health risks of improper disposal. During medication reviews, we remind patients to check their supplies at home and bring back anything they no longer need.

**Interviewer:** And have you observed any changes in patient attitudes or behaviors regarding medicine returns and disposals over time?

**Pharmacist:** Definitely. Over the years, I've noticed that more patients are becoming aware of the issue and are willing to return their unused medicines. However, there's still room for improvement. Some patients find it inconvenient or simply forget, which is why we need to make the process as easy and accessible as possible.

**Interviewer:** In terms of collaboration, do you collaborate with other healthcare professionals or organizations to address issues related to medicine disposal and returns?

**Pharmacist:** Yes, collaboration is key. We work with local GP practices, nursing homes, and nonprofit organizations to promote safe disposal practices. For example, we've partnered with environmental groups to run awareness campaigns. These partnerships help us reach a wider audience and drive positive change.

**Interviewer:** Are there any resources or support systems available to assist community pharmacies in improving their management of returned medicines and medicine wastage?

**Pharmacist:** There are some resources available, such as guidance documents from the Pharmaceutical Society and training programs on waste management. However, I think more support is needed, particularly in terms of funding and clear, enforceable policies. It would also be helpful to have a standardized medicines return service across all pharmacies.

**Interviewer:** Perfect. Can you share any innovative strategies or initiatives your pharmacy has implemented to enhance the management of returned medicines and reduce medicine wastage?

**Pharmacist:** One initiative we've introduced is a medication check-up service where patients can bring in all their medications for a review. This helps identify unused or expired medicines and provides an opportunity to educate patients about safe disposal. We've also started using digital reminders to prompt patients to check their medication supplies at home.

**Interviewer:** And what do you envision as potential future developments or improvements in this area within pharmacy practice?

**Pharmacist:** I'd like to see a more coordinated approach at the national level with clear guidelines and funding for medicines return services. I also think technology could play a bigger role—for example, apps that remind patients to return unused medicines or track their medication supplies. Ultimately, I believe community pharmacies should be seen as central hubs for safe medicine disposal, supported by both the government and pharmaceutical companies.

**Interviewer:** Perfect. And is there anything else you'd like to add or discuss regarding the management of returned medicines, wastage, and safe disposal in community pharmacies?

**Pharmacist:** I'd like to emphasize how important this issue is for public health and the environment. As pharmacists, we have a responsibility to lead by example and support government initiatives. However, we also need more clarity and resources to do this effectively. I'm hopeful that with greater collaboration and awareness, we can make significant progress in this area.

**Interviewer:** Perfect. Thank you so much for your participation in this interview. Would you be willing to provide further input or clarification if required in the future?

**Pharmacist:** Absolutely. I'd be happy to help in any way I can. Thank you for the opportunity to discuss this important topic.

**Interviewer:** Lovely. Thank you so much.

**Pharmacist:** Thank you.

**P 9**

**Interviewer:** So, just to start, can you provide some background on your experience working in community pharmacies in Northern Ireland?

**Pharmacist:** I've been a community pharmacist for eight years now, working in a busy pharmacy. Over the years, I've seen a lot of changes in the profession, especially with increasing demands on our time and resources. It's a challenging but rewarding role, though there are definitely areas where I feel we could do better.

**Interviewer:** And how long have you been involved in pharmacy practice, and what motivated you to pursue this career path?

**Pharmacist:** I've always been interested in healthcare and wanted a career where I could make a direct impact on people's lives. Pharmacy offered that balance of science and patient care. However, I'll admit that the reality of the job, especially the administrative and regulatory burdens, has been more challenging than I expected.

**Interviewer:** Can you describe the current procedures in place for managing returned medicines in your community pharmacy?

**Pharmacist:** We have a system in place where patients can return unused or expired medicines to us. These are stored in a secure bin until they are collected by a waste disposal company. However, the process isn't perfect. We often get patients bringing in large quantities of medicines, which can be difficult to store safely, especially in a small pharmacy like ours.

**Interviewer:** And what measures are typically taken to minimize medicine wastage in your pharmacy?

**Pharmacist:** We try to educate patients about only ordering what they need, but it's an uphill battle. Some patients are on repeat prescriptions and don’t always check what they already have at home. We also work with GPs to ensure prescriptions are appropriate, but there’s still a lot of wastage. It’s frustrating because it feels like we’re constantly fighting against the system.

**Interviewer:** What challenges do you encounter regarding the management of returned medicines and minimizing wastage in your pharmacy?

**Pharmacist:** One of the biggest challenges is the sheer volume of returned medicines we handle. It's not uncommon for patients to bring in bags full of medications, some of which are expensive or in short supply. It's a waste of resources, but there’s not much we can do about it once the medicines have been dispensed.

Another issue is the lack of patient awareness. Many people don’t realize they can return medicines to the pharmacy, so they end up hoarding them at home or disposing of them improperly. Even when they do bring them back, it’s often because they’ve been told to by a family member or carer, not because they understand the importance of safe disposal.

**Interviewer:** Are there any concerns or issues related to the safe disposal of unused or expired medications?

**Pharmacist:** The regulatory requirements can be quite burdensome. We have to ensure that returned medicines are stored securely and disposed of by licensed contractors, which adds to our workload. There is also a lot of paperwork involved, and it’s not always clear what the guidelines are.

For example, we’re not allowed to reuse any returned medications, even if they’re unopened and in perfect condition. I understand the safety concerns, but it feels like a missed opportunity to reduce waste, especially with the current pressures on the healthcare system.

**Interviewer:** Are there any existing policies or initiatives at the local or national level that influence your pharmacy’s practices in this regard?

**Pharmacist:** We try to educate patients through posters, leaflets, and conversations at the counter, but it’s hard to get the message across. Many patients are more focused on getting their prescriptions filled than on what happens to their unused medicines.

**Interviewer:** In terms of collaboration, do you collaborate with any other healthcare professionals or organizations to address issues related to medicine returns and disposal?

**Pharmacist:** We work with local nursing homes to an extent, but there’s definitely room for more collaboration. For example, it would be helpful if GPs could remind patients to return unused medicines during consultations.

**Interviewer:** Can you share any innovative strategies or initiatives your pharmacy has implemented to enhance the management of returned medicines and reduce medicine wastage?

**Pharmacist:** The big thing we’ve introduced is a "Medication Honesty Day" once a year, where we encourage patients to bring in their unused medicines. It’s been quite successful in raising awareness, but it’s only a small step.

**Interviewer:** What do you envision as potential future developments or improvements in this area within pharmacy practice?

**Pharmacist:** I’d like to see more support from the government, both in terms of funding and clear guidelines. It would also be helpful to have a standardized system for managing returned medicines across all pharmacies.

Another area for improvement is patient education. We need more public awareness campaigns to highlight the importance of safe medicine disposal and the role of pharmacies in this process. Ultimately, I think community pharmacies should be seen as key players in medicines management, but we need the resources and support to make that happen.

**Interviewer:** And just to close, is there anything else you’d like to add or discuss regarding the management of returned medicines, wastage, and safe disposal in community pharmacies?

**Pharmacist:** I think it’s important to recognize that this is a complex issue that requires a coordinated approach. Pharmacies are doing their best, but we can’t solve the problem on our own. We need more support from the government, better collaboration with other healthcare professionals, and greater awareness among patients.

It’s also worth noting that this issue has implications for patient safety and the sustainability of the healthcare system. By addressing medicine wastage and improving disposal practices, we can not only protect the environment but also ensure that resources are used more effectively.

**Interviewer:** Perfect. Thank you so much for your participation in this interview. Would you be willing to provide further input or clarification if required in the future?

**Pharmacist:** Absolutely. I would be happy to help.

**Interviewer:** Lovely. Thank you so much for your time.

**Pharmacist:** Thank you.

**P 10**

**Interviewer:** So, just to start, can you provide some background on your experience working in community pharmacies in Northern Ireland?

**Pharmacist:** I've been a community pharmacist for nearly 15 years, working in a variety of settings, from small rural pharmacies to larger urban ones. Over the years, I've seen the role of the pharmacist expand significantly, particularly in terms of patient care and public health initiatives. It's been a fulfilling career, but it's also highlighted areas where we need to improve, especially around medicines management.

**Interviewer:** And how long have you been involved in pharmacy practice, and what motivated you to pursue this career path?

**Pharmacist:** I've always been drawn to healthcare and the idea of making a tangible difference in people's lives. Pharmacy offered the perfect blend of science and patient interaction. What really motivates me now is the opportunity to innovate and find better ways to support patients and improve public health outcomes.

**Interviewer:** In terms of current procedures, what procedures do you have in place for managing returned medicines in your community pharmacy?

**Pharmacist:** We have a secure medicines return bin where patients can drop off unused or expired medications. These are collected by a licensed waste disposal company on a regular basis. However, the process isn't without its challenges. For example, we often receive large quantities of returned medicines, which can be difficult to manage, especially in smaller pharmacies with limited storage space.

**Interviewer:** What measures are typically taken to minimize medicine wastage in your pharmacy?

**Pharmacist:** We take a proactive approach to minimize wastage. For example, we encourage patients to only order the medications they need and to check their supplies at home before requesting repeats. We also work closely with prescribers to ensure prescriptions are appropriate and tailored to the patient's needs, which helps reduce overprescribing.

**Interviewer:** What challenges do you encounter regarding the management of returned medicines and minimizing medicine wastage in your pharmacy?

**Pharmacist:** One of the biggest challenges is the lack of patient awareness and engagement. Many patients don't realize they can return unused medicines to the pharmacy, so they end up hoarding them at home or disposing of them improperly. Even when they do bring them back, it's often because they've been told to by a family member or carer, not because they understand the importance of safe disposal.

Another issue is the impact of medication shortages. When patients can't get the medications they need, they may stockpile them when they become available, which leads to wastage when they no longer need them. This is a growing problem that needs to be addressed at a systemic level.

**Interviewer:** And in terms of policy, how do you navigate regulatory requirements and guidelines related to the management and disposal of returned medicines and expired medications?

**Pharmacist:** Regulatory requirements can be quite complex and sometimes difficult to interpret. We do our best to comply, but it's not always clear what's expected of us. For example, there are strict rules about how returned medicines should be stored and disposed of, but the guidelines aren't always straightforward.

**Interviewer:** Are there any existing policies or initiatives at the local or national level that influence your pharmacy's practices in this regard?

**Pharmacist:** There are some government initiatives aimed at promoting safe medicine disposal, but they are not always well publicized or supported. For example, the take-back scheme is a good idea, but it's not widely known among patients. I think there needs to be more investment in public awareness campaigns to make these initiatives effective.

**Interviewer:** In terms of patient engagement, how do you educate patients about the importance of returning unused medicines and proper disposal methods?

**Pharmacist:** We use a combination of posters, leaflets, and one-on-one conversations to educate patients. We also try to incorporate this message into our medication reviews, where we remind patients to check their supplies at home and bring back anything they don't need. However, I think we could do more in terms of patient engagement. For example, it would be helpful to have more resources available, such as videos or social media campaigns to reach a wider audience.

**Interviewer:** In terms of collaboration, do you collaborate with other healthcare professionals or organizations to address issues related to medicine returns and disposal?

**Pharmacist:** We do collaborate with local GPs and nursing homes to some extent, but there's definitely room for more coordination. For example, it would be helpful if GPs could remind patients to return unused medicines during consultations.

**Interviewer:** Can you share any innovative strategies or initiatives your pharmacy has implemented to enhance the management of medicine returns and reduce medicine wastage?

**Pharmacist:** I think the main thing we try to do is talk to patients when we notice a pattern of frequent medicine returns. If we see someone bringing back large amounts of medication at the end of every month, we have a conversation to understand why and see if there’s anything we can do to help. However, we don't get a lot of training or opportunities to brainstorm new initiatives. I think this is partly because patients themselves are not fully aware of the issue of medicine wastage.

**Interviewer:** What do you envision as potential future developments or improvements in this area within pharmacy practice?

**Pharmacist:** I'd like to see more support from the government, both in terms of funding and clear guidelines. It would also be helpful to have a standardized system for managing returned medicines across all pharmacies.

Another area for improvement is patient education. We need more public awareness campaigns to highlight the importance of safe medicine disposal and the role of pharmacies in this process. Ultimately, I think community pharmacies should be seen as key players in medicines management, but we need the resources and support to make that happen.

**Interviewer:** Is there anything else you'd like to add or discuss regarding the management of returned medicines, wastage, and disposal in community pharmacies?

**Pharmacist:** I think it's important to recognize that this is a complex issue that requires a coordinated approach. Pharmacies are doing their best, but we can't solve the problem on our own. We need more support from the government, better collaboration with other healthcare professionals, and greater awareness among patients.

It's also worth noting that this issue has implications for patient safety and the sustainability of the healthcare system. By addressing medicine wastage and improving disposal practices, we can not only protect the environment but also ensure that resources are used more effectively.

**Interviewer:** Thank you so much for your participation in this interview. Would you be willing to provide further clarification if required in the future?

**Pharmacist:** Absolutely. I’d be happy to help. Thank you for giving me the opportunity to share my thoughts.

**Interviewer:** Lovely. Thank you so much for your time.

**Pharmacist:** Thank you.

**P 11**

**Interviewer**: So just to start, can you provide some background on your experience working in community pharmacies in Northern Ireland?

**Participant:** Absolutely. I've been working as a community pharmacist here in Northern Ireland for about 12 years now. I started out as a pre-registration pharmacist in a small independent pharmacy, and I've since worked in a few different settings, including larger chain pharmacies. It's been a really rewarding career, and I've had the chance to build strong relationships with patients and the local community.

**Interviewer:** And how long have you been involved in pharmacy practice? What motivated you to pursue this career?

**Participant:** Well, I've been in pharmacy practice for over a decade, as I mentioned. What really drew me to this career was the blend of science and patient care. I've always been passionate about helping people, and pharmacy allows me to do that in a very practical way. Plus, I love the problem-solving aspect of it—whether it's finding the right medication for a patient or advising them on managing their health.

**Interviewer:** Very good. And can you just describe the current procedures in place for managing returned medicines in your community pharmacy?

**Participant:** Sure. When patients return medicines to us, we follow a strict protocol. First, we separate the returned medications into categories: controlled drugs, non-controlled drugs, and hazardous waste like cytotoxic medications. Controlled drugs are handled with extra care due to legal requirements. All returned medicines are then placed in secure bins provided by a licensed waste management company, which collects and disposes of them safely. We also keep a record of all returned medications for auditing purposes.

**Interviewer:** Very good. And what measures are typically taken to minimize medicine wastage in your pharmacy?

**Participant:** We try to tackle wastage at the source. For example, we encourage patients to only order what they need, especially for repeat prescriptions. We also work closely with GPs to ensure prescriptions are appropriate and up to date. Additionally, we do regular stock checks to avoid over-ordering and expiries. For patients on long-term medications, we advise them to have their medications reviewed to make sure they're still needed and being used correctly.

**Interviewer:** Very good. And what challenges do you encounter regarding the management of returned medicines and minimizing medicine wastage at your pharmacy?

**Participant:** One of the biggest challenges is patient awareness. Many people don't realize they can return unused medicines to us, so they end up hoarding them at home or throwing them in the bin, which isn't safe. Even when they do return them, it's often a large quantity, which is a waste of resources. Another issue is the cost—safe disposal isn't cheap, and it's something we have to factor into our budget.

**Interviewer:** And are there any concerns or issues related to the safe disposal of unused or expired medications?

**Participant:** The environment. If medicines aren't disposed of properly, they can end up in waterways or soil, which is harmful. There's also the risk of accidental ingestion, especially if medicines are thrown in household waste. We always stress to patients that bringing them back to us is the safest option.

**Interviewer:** Very good. And then how do you navigate regulatory requirements and guidelines related to the management and disposal of returned and expired medications?

**Participant:** We follow the guidelines set by the Department of Health. These cover everything from how to handle controlled drugs to the correct labeling of waste bins. The guidelines can be a bit confusing at times, and there can be a lot of confusion between pharmacists about what the best procedure is regarding medicine returns. It's a lot to keep track of, but it's essential for patient safety and legal compliance.

**Interviewer:** Very good. Are there any existing policies or initiatives at the local or national level that influence your pharmacy's practices in this regard?

**Participant:** Yeah, honestly, now that I think about it, I can't really remember any schemes that the government has issued regarding medicine disposal and returns, which is quite unfortunate. I think it would be a good idea if the government launched a campaign about this to really help raise awareness and make our job easier.

**Interviewer:** Very good. And in terms of patient engagement, how do you educate patients about the importance of returning unused medications and proper disposal methods?

**Participant:** We use a few different approaches. For example, we have posters and leaflets in the pharmacy, and we often include sticker reminders on prescription bags if a patient is using a lot of medications. When dispensing, we'll sometimes have a quick chat with patients about returning unused medicines, especially if they're on a new medication or have had a change in their treatment.

**Interviewer:** Good. And have you observed any changes in patient attitudes or behaviors regarding medicine returns and disposal over time?

**Participant:** Definitely. Over the years, I've noticed more patients are aware of the issue and are willing to return their unused medicines these days. That said, there's still a long way to go. Some patients still don't see it as a priority, so we need to keep pushing the message.

**Interviewer:** Very good. In terms of collaboration, do you collaborate with other healthcare professionals or organizations related to medicine returns and disposal?

**Participant:** No, not really in my experience. The only one I can think of is with nursing home staff. We provide them with information on how to store, return, and dispose of any of their residents' medications, especially when it comes to controlled drugs. But other than that, I can't really think of anyone else.

**Interviewer:** And are there any resources or support systems available to assist community pharmacies in improving their management of returned medicines and minimizing wastage?

**Participant:** The Health and Social Care Board provides guidance and resources, and organizations like Community Pharmacy NI offer training and support. We also have access to online tools and templates for things like the controlled drugs destruction template.

**Interviewer:** Very good. Can you share any innovative strategies or initiatives your pharmacy has implemented to enhance the management of returned medicines and reduce medicine wastage?

**Participant:** One thing we did a couple of years ago was change the prescription bags from plastic to paper bags. It's a simple idea, but I think it is a really good step toward a smaller eco-footprint. It's a small change, but a step in the right direction.

**Interviewer:** Very good. And what do you envision as potential future developments or improvements in this area within pharmacy practice?

**Participant:** I think technology will play a big role. For example, smart packaging that tracks when a medication is opened or expired could help reduce waste. I also hope to see more integrated systems between pharmacies, GPs, and hospitals to streamline medication management. And of course, continued patient education will be key.

**Interviewer:** Perfect. And is there anything else you'd like to add or discuss?

**Participant:** Just that it's a team effort—pharmacists, patients, and healthcare providers all have a role to play. The more we work together, the better we can tackle these issues. It's not just about saving money; it's about protecting the environment and ensuring patient safety.

**Interviewer:** Lovely. Thank you so much for your participation in this interview. Would you be willing to provide further clarification if required in the future?

**Participant:** Happy to help anytime. It's an important topic, and I'm always happy to share my experience.

**Interviewer:** Lovely. Thank you so much.

**P 12**

**Interviewer:** So just to start, can you provide some background on your experience working in community pharmacies in Northern Ireland?

**Participant:** Yes, sure. I've been working as a community pharmacist here for about three years now. I started in a busy urban pharmacy and later moved to a smaller rural setting. It's been a mix of challenges and rewards, especially when it comes to managing patient needs and navigating the complexities of pharmacy practice.

**Interviewer:** And how long have you been involved in pharmacy practice? What motivated you to pursue this career path?

**Participant:** I've been in pharmacy practice since I qualified three years ago. What drew me to this career was the opportunity to make a real difference in people's lives. I've always been fascinated by how medicines work and how they can improve health outcomes. Plus, I enjoy the day-to-day interactions with patients. It keeps things interesting.

**Interviewer:** Can you describe the current procedures in place for managing returned medicines in your pharmacy?

**Participant:** At the moment, when patients return medicines, we separate them into categories like solids, liquids, and controlled drugs. We then store them in designated bins provided by a waste management company, which collects them every week. It's a fairly straightforward process, but it does require careful handling, especially with controlled drugs.

**Interviewer:** And what measures are typically taken to minimize medicine wastage in your pharmacy?

**Participant:** We try to minimize waste by encouraging patients to only order what they need. For example, we often ask if they've checked their medicine cabinet before ordering a repeat prescription. We also do regular checks to avoid over-ordering and expiries. However, it's not always easy to predict demand, so some wastage is inevitable.

**Interviewer:** And what challenges do you encounter regarding the management of returned medicines and minimizing medicine wastage in your pharmacy?

**Participant:** One of the biggest challenges is the lack of clear, consistent guidelines. The rules around handling and disposing of returned medicines can be quite confusing, especially when it comes to controlled drugs. It feels like there's a lot of grey areas, and I often find myself double-checking regulations to make sure we're compliant. It's time-consuming and stressful at times.

**Interviewer:** Are there any concerns or issues related to the safe disposal of unused or expired medicines?

**Participant:** Absolutely. The main concern is ensuring that medicines are disposed of safely to avoid environmental harm or accidental ingestion. However, not all patients are aware of the risks, so we often find ourselves dealing with large quantities of expired or unused medications that could have been avoided.

**Interviewer:** And how do you navigate regulatory requirements and guidelines related to the management and disposal of returned and expired medications?

**Participant:** Honestly, it's a bit of a minefield. The guidelines can be quite complex and sometimes contradictory, especially when you're dealing with different types of waste. I often have to refer back to the PSNI's guidance or consult with colleagues to make sure we're doing things correctly. It's not always straightforward, and I think clearer, more user-friendly guidelines would be a huge help.

**Interviewer:** And are there any existing policies or initiatives at the local or national level that influence your pharmacy's practices in this regard?

**Participant:** I do believe I have seen some, but I can't remember off the top of my head. However, I feel like there's a lack of coordination at the local level. For example, we don't have a clear system for working with GPs or other healthcare providers to reduce wastage. It feels like we're all working in silos, which isn't ideal.

**Interviewer:** And in terms of patient engagement, how do you educate patients about the importance of returning unused medications and proper disposal?

**Participant:** We use a mix of approaches, like posters, leaflets, and one-on-one conversations when dispensing medications. We’ll often remind patients to only order what they need and to bring back any unused medicines. However, I think there's room for improvement in terms of patient education. Many people still don't fully understand the risks of hoarding or improperly disposing of medications.

**Interviewer:** Have you observed any changes in patient attitudes or behavior regarding medicine returns and disposal?

**Participant:** There's been some improvement, but it's slow. More patients are aware of the issue, but I still see a lot of unnecessary wastage. I think part of the problem is that people don't see it as a priority, so we need to keep pushing the message.

**Interviewer:** And in terms of collaboration, do you collaborate with other healthcare professionals to address issues related to medicine returns and disposal?

**Participant:** Not really, to be honest. We tend to operate independently, which I think is a missed opportunity. For example, it would be great to work more closely with GPs to ensure that prescriptions are appropriate and to identify patients who might be at risk of over-ordering. But at the moment, there's no formal system in place for that kind of collaboration.

**Interviewer:** And are there any resources or support systems available to assist community pharmacies in improving their management of returned medicines and medicine wastage?

**Participant:** There are some resources available, like guidance documents from the government, but I find them quite dense and difficult to navigate. I think more practical support, like training sessions or toolkits, would be really helpful.

**Interviewer:** Can you share any innovative strategies or initiatives your pharmacy has implemented to enhance the management of returned medicines and reduce medicine wastage?

**Participant:** We've started using a medicine return box where patients can drop off unused medications anonymously. It's been quite successful in encouraging returns, but it's still a small-scale initiative. I'd love to see more innovative approaches.

**Interviewer:** And what do you envision as potential future developments or improvements in this area within pharmacy practice?

**Participant:** I think there's huge potential for better integration between pharmacies, GPs, and other healthcare providers. For example, a shared digital system could help us track medication usage and identify wastage hotspots. I'd also like to see more investment in patient education and clearer, more accessible guidelines for pharmacists.

**Interviewer:** Very good. And just to close, is there anything else you’d like to add or discuss regarding this topic?

**Participant:** Just that it's a complex issue that requires a coordinated approach. At the moment, it feels like we're all working in isolation, which isn't helping anyone. I'd love to see more collaboration and support from both regulators and other healthcare providers.

**Interviewer:** Thank you so much for your participation. Would you be willing to provide further clarification if needed in the future?

**Participant:** Absolutely, I'd be happy to help. It's an important issue, and I'm always keen to share my perspective.

**Interviewer:** Lovely, thank you so much for your time.

**P 13**

**Interviewer:** So, does the start—can you provide some background on your experience working in community pharmacies in Northern Ireland?

**Participant:** Sure. I've been working in community pharmacy for ten years now. It's a busy job, and every day brings a new challenge. You get to know your regular patients, which is great, but it can also be frustrating when you see the same issues over and over—people not taking their medicines properly, over-ordering, and then bringing bags of unused medications back. We do our best, but it often feels like we're just firefighting rather than actually solving the problem.

**Interviewer:** And how long have you been involved in pharmacy practice, and what motivated you to pursue this career path?

**Participant:** I liked the idea of being in the community rather than in a hospital. Pharmacy seemed like the perfect balance of science and patient care. I wanted to make a difference in people's lives, but honestly, some days it feels like all I do is check prescriptions.

**Interviewer:** And could you describe the current procedures in place for managing returned medicines in your pharmacy?

**Participant:** Yes, well, patients return medicines, and we have special bins for disposal. Controlled drugs need to be denatured before disposal, which is a bit of a hassle, but we have to follow strict regulations. A waste disposal company collects everything regularly. The system works, but the volume of returns can be overwhelming, and we just don't have enough space in the pharmacy for all of it.

**Interviewer:** And what measures are typically taken to minimize medicine wastage in your pharmacy?

**Participant:** We try to educate patients about only ordering what they need, but, let's be honest, some don’t listen. Some stockpile medicines just in case, and then months later, they bring them back unopened. We also try to check with patients before dispensing large repeat prescriptions, but we don't have much influence over what GPs prescribe, and sometimes it's hard to get through to patients who think they need everything just because it's on their script.

**Interviewer:** Yeah, and what challenges do you encounter regarding the management of returned medicines and minimizing medicine wastage?

**Participant:** The biggest problem is storage. Some days, it feels like half the town has decided to clear out their medicine cabinets, and we just don't have the space to keep all those returns until collection day.

**Interviewer:** And are there any concerns or issues related to the safe disposal of unused or expired medications?

**Participant:** People still throw tablets in the bin or flush them down the toilet, which is awful for the environment. We try to tell them to bring everything back to us, but not everyone listens. And controlled drugs—those are a nightmare. We have to make sure they're destroyed properly so they don't end up in the wrong hands.

**Interviewer:** And how do you navigate regulatory requirements and guidelines related to the management and disposal of returned medicines and expired medicines?

**Participant:** The paperwork is ridiculous. There are so many rules, and every time a new regulation comes in, it adds to the workload.

**Interviewer:** Are there any existing policies or initiatives at the local or national level that influence your pharmacy's practices in this regard?

**Participant:** The government has medicine waste campaigns, but they don't really tackle the root of the problem. The policies are strict—once a medicine is dispensed, it can't be reused—but there's no real effort to prevent waste in the first place. We could do with more flexibility, especially for unopened medications that could go to care homes or charities instead of being destroyed.

**Interviewer:** And in terms of the patient, how do you educate patients about the importance of returning unused medications and proper disposal methods?

**Participant:** Mostly through one-on-one conversations at the counter. If a patient brings in a big bag of returns, we'll have a chat about why they have so much left over and try to stop it from happening again.

**Interviewer:** And have you observed any changes in patient attitudes or behaviors regarding medicine returns and disposal over time?

**Participant:** Some people are more aware of the environmental impact now, but there's still plenty who hoard medicines because they don’t want to bother the doctor by asking for another prescription later. And some just don’t understand why they can't return unopened packs for someone else to use.

**Interviewer:** And do you collaborate with other healthcare professionals or organizations to address issues related to medicine returns and disposal?

**Participant:** Not really. I mean, we try to flag it with GPs if a patient has loads of medication, but communication isn't great. It would help if GPs reviewed prescriptions more often and reminded patients to only order what they actually need.

**Interviewer:** And are there any resources or support systems available to assist community pharmacies in improving their management of returned medicines and minimizing wastage?

**Participant:** It's up to individual pharmacies to figure out the best way to handle returns. Some pharmacies are trying things like prescription synchronization to reduce over-ordering, but there's no real system-wide approach.

**Interviewer:** And can you share any innovative strategies or initiatives your pharmacy has implemented to enhance the management of returned medicines and reduce medicine wastage?

**Participant:** We check with patients before dispensing repeats, asking if they really need everything. Some patients appreciate it, but others just say, "I'll take it anyway."

**Interviewer:** And what do you envision as potential future developments or improvements in this area within pharmacy practice?

**Participant:** If pharmacies had real-time prescription tracking, we could catch over-ordering before it happens. And honestly, there should be a way to redistribute unopened medicines instead of destroying them, but I imagine the regulations around that would be a nightmare to change.

**Interviewer:** And just to close, is there anything else you’d like to add or discuss regarding the management of returned medicines, wastage, and disposal?

**Participant:** Pharmacies are doing their best, but we need more support. Patients need better education, GPs need to review prescriptions more carefully, and the whole system needs to be less rigid about what we can and can’t reuse. Otherwise, we're just going to keep throwing perfectly good medicines in the bin.

**Interviewer:** Perfect. Thank you so much for your insights into this interview. Would you be open to providing further input if needed in the future?

**Participant:** Sure. Hopefully, something actually changes one day.

**Interviewer:** Lovely. Thank you so much.

**Participant:** Thank you.

**P 14**

**Interviewer:** So, to start, can you provide some background on your experience working in community pharmacies in Northern Ireland?

**Participant:** I've been in community pharmacy for over a decade. I started off thinking it would be a rewarding mix of science and patient care, and while that’s true, the reality is that a lot of my time is spent dealing with issues like medicine waste, stock management, and regulatory red tape. It’s not always the patient-facing role I envisioned when I first got into this, but I do still enjoy being able to support my community.

**Interviewer:** And how long have you been involved in pharmacy practice, and what motivated you to pursue this career?

**Participant:** It was a combination of wanting to help people and the problem-solving aspect of medicine. I liked the idea of working directly with the public rather than being in a lab all day, but honestly, what they don’t tell you is how much of your time is spent dealing with bureaucracy rather than patient care. The amount of paperwork and regulations we have to navigate can be overwhelming.

**Interviewer:** And can you describe the current procedures in place for managing returned medicines in your community pharmacy?

**Participant:** We have bins for medicines that get collected by a waste disposal company. That part is pretty straightforward, but the challenge is what leads to all these returns in the first place. Patients often bring back huge amounts of medicines—sometimes entire months’ worth of prescriptions that were never touched. It’s frustrating because once those medicines leave the pharmacy, there’s nothing we can do but throw them away, even if they’re unopened and perfectly safe.

**Interviewer:** And what measures are typically taken to minimize medicine wastage in your pharmacy?

**Participant:** We do our best, but it often feels like we’re fighting an uphill battle. We try to flag when patients are over-ordering, but the repeat prescription system doesn’t always help. GPs sometimes put patients on automatic repeats, and by the time we intervene, they’ve already collected more medicine than they need. And let’s be honest, some patients just don’t listen when we advise them to only order what they actually require.

**Interviewer:** What challenges do you encounter regarding the management of returned medicines and minimizing medicine wastage in your pharmacy?

**Participant:** A big issue is returned medicines for elderly or vulnerable patients. It’s not unusual for a relative to come in with a bag of medication saying the patient has passed away or moved into care. It’s heartbreaking, but also frustrating, because in some cases, the patient was being prescribed way more than they actually needed in the first place. There’s a massive lack of medication reviews for people in care homes or those with complex conditions.

**Interviewer:** And are there any concerns or issues related to the safe disposal of unused or expired medicines?

**Participant:** Another big issue is storage. We’re a small pharmacy, and when we get a large volume of returned medicines, we quickly run out of space to hold them before collection. If we don’t stay on top of it, our back room starts looking like a stockroom of waste. And sometimes, collection schedules aren’t as regular as they should be, which just adds to the problem.

**Interviewer:** And how do you navigate regulatory requirements and guidelines related to the management and disposal of returned medicines and expired medications?

**Participant:** Honestly, with difficulty. The regulations are necessary—I get that—but they’re not always practical. The amount of paperwork involved in handling controlled drug returns is ridiculous. If a patient brings back a controlled drug, we have to document every detail, denature it in a special kit, and then store it separately until disposal. It takes up time and resources that we just don’t have.

**Interviewer:** Are there any existing policies or initiatives at the local or national level that influence your pharmacy’s practices in this regard?

**Participant:** There needs to be more flexibility in how we handle returns. If a patient brings back a completely sealed box of medication that was dispensed a week ago, why should we be forced to destroy it? I understand the safety concerns, but surely there should be a way to check and redistribute certain medicines rather than just sending everything straight to waste. It feels like an unnecessary drain on NHS resources.

**Interviewer:** And in terms of the actual patient, how do you educate patients about the importance of returning unused medications and proper disposal methods?

**Participant:** It’s one of the most frustrating parts of the job. We tell people at the counter, we have posters up, we hand out leaflets—but at the end of the day, it comes down to changing long-standing habits. Some patients just aren’t engaged with their own medication use. They pick up prescriptions out of routine, even if they don’t actually need them anymore, and then we see the same medications coming back months later in bin bags.

**Interviewer:** And have you observed any changes in patient attitudes or behaviors regarding medicine returns and disposal over time?

**Participant:** A little—some people are more aware of environmental issues, but not nearly enough. A lot of people still think it’s fine to throw pills in the bin or flush them down the sink. And then you get others who genuinely believe they’re helping by bringing back unused medicines, only for our staff to explain that we can’t reuse them. Patients get frustrated, and so do we.

**Interviewer:** And do you collaborate with other healthcare professionals or organizations to address issues related to medicine returns and disposal?

**Participant:** If GPs could check in more often with patients about whether they actually need repeat prescriptions, it would make a huge difference. But in reality, GPs are stretched thin, pharmacies are overstretched, and medicine waste just keeps piling up.

**Interviewer:** Are there any resources or support systems available to community pharmacies in improving their management of returned medicines and wastage?

**Participant:** There are guidelines, but they don’t go far enough. It’s mostly down to individual pharmacies to figure out how to handle the logistics. Some pharmacies have started using digital tools to track medicine use and flag issues early, but there’s no standardized system. A centralized NHS-led approach would be much more effective.

**Interviewer:** And can you share any innovative strategies or initiatives your pharmacy has implemented to enhance the management of returned medicines and reduce medicine wastage?

**Participant:** We’ve been trialing a system where we send a text to remind patients about their repeats before dispensing, to check if they still need everything. It’s a small step, but it has helped cut down on some unnecessary prescriptions. But again, there’s no universal approach.

**Interviewer:** And what do you envision as future developments or improvements in this area within pharmacy practice?

**Participant:** I think there needs to be better coordination across the board. Pharmacies, GPs, and regulators need to work together to ensure patients aren’t being given more medicine than they need. And also, there needs to be a serious discussion about allowing the safe redistribution of unopened medicines. Destroying perfectly good medication while the NHS struggles for resources makes no sense.

**Interviewer:** And is there anything else you’d like to add or discuss regarding the management of returned medicines, wastage, and disposal in community pharmacies?

**Participant:** It’s a frustrating cycle. Pharmacies are doing their best, but without proper support, we’re just managing the problem rather than solving it. Patients need better education, GPs need to review prescriptions more often, and the government needs to rethink some of these rigid policies. Until then, we’ll just keep filling up our waste bins and wondering how much of it could have been avoided.

**Interviewer:** Thank you so much for your insight and participation in this interview. Would you be willing to provide further input or clarification if needed in the future?

**Participant:** Of course.

**Interviewer:** Lovely, thank you so much for your time.

**Participant:** Thank you.

**P 15**

**Interviewer:** So, to start, can you provide some background on your experience working in community pharmacies in Northern Ireland?

**Participant:** I've been in community pharmacy for 16 years now, working in both urban and rural settings. It’s been a really varied experience—no two days are the same, which is part of what I love about the job. I’ve seen a lot of changes over the years, especially when it comes to managing medicines and dealing with waste.

**Interviewer:** And how long have you been involved in pharmacy practice, and what motivated you to pursue this career path?

**Participant:** I've been in pharmacy practice since I qualified a decade ago. I was drawn to pharmacy because it’s such a hands-on way to help people. I’ve always been interested in health and science, and pharmacy lets me combine those interests while making a real difference in people’s lives.

**Interviewer:** Perfect, and can you describe the current procedures in place for managing returned medicines in your community pharmacy?

**Participant:** When patients return medicines, we sort them into categories—tablets, liquids, inhalers, and so on—and place them in specific bins for collection by a licensed waste disposal company. Controlled drugs are handled separately because of the stricter regulations around them. We also keep a log of what’s returned, especially for controlled drugs, to ensure everything is accounted for.

**Interviewer:** Perfect, and what measures are typically taken to minimize medicine wastage in your pharmacy?

**Participant:** We try to tackle wastage at the source by encouraging patients to only order what they need. For example, we’ll ask if they’ve checked that they are definitely out of their medication before ordering a repeat prescription. We also do regular stock checks to avoid overordering and expiries. However, it’s not always easy to predict demand, so some wastage is inevitable.

**Interviewer:** And what challenges do you encounter regarding the management of returned medicines and medicine wastage in your pharmacy?

**Participant:** One of the biggest challenges is the lack of clear, consistent guidelines. The rules around handling and disposing of returned medicines can be quite confusing, especially when it comes to controlled drugs. It feels like there’s a lot of gray area, and I often find myself double-checking regulations to make sure we’re compliant. It’s time-consuming and stressful at times.

**Interviewer:** And are there any concerns or issues related to the safe disposal of unused or expired medications?

**Participant:** Absolutely. The main concern is ensuring that medicines are disposed of safely to avoid environmental harm or patient safety risks. However, not all patients are aware of these risks, so we often find ourselves dealing with large quantities of expired or unused medications that could have been avoided.

**Interviewer:** And how do you navigate regulatory requirements and guidelines related to the management and disposal of returned medicines and expired medications?

**Participant:** Honestly, it’s a bit of a challenge. The guidelines can be quite complex and sometimes contradictory, especially when dealing with different types of waste. I often have to consult with colleagues to make sure we’re doing things correctly. It’s not always straightforward, and I think clearer, more user-friendly guidelines would be a huge help.

**Interviewer:** And are there any existing policies or initiatives at the local or national level that influence your pharmacy’s practices in this regard?

**Participant:** There are some national campaigns, but I feel like there’s a lack of coordination at the local level. For example, we don’t have a clear system for working with GPs or other healthcare providers to reduce wastage. It feels like we’re all working in silos, which isn’t ideal.

**Interviewer:** And in terms of the patient, how do you educate patients about the importance of returning unused medicines and proper disposal methods?

**Participant:** We mainly use one-on-one conversations when dispensing medications. We’ll often remind patients to only order what they need and to bring back any unused medicines. However, I think there’s room for improvement in terms of patient education—many people still don’t fully understand the risks of hoarding or improperly disposing of medications.

**Interviewer:** And have you observed any changes in patient attitudes or behaviors regarding medicine returns and disposal over time?

**Participant:** More patients are aware of the issue, but I still see a lot of unnecessary wastage. I think part of the problem is that people don’t see it as a priority, so we need to keep pushing the message.

**Interviewer:** And do you collaborate with other healthcare professionals or organizations to address issues related to medicine returns and disposal?

**Participant:** Not really, to be honest. We tend to operate independently, which I think is a missed opportunity. For example, it would be great to work more closely with GPs to ensure that prescriptions are appropriate and to identify patients who might be at risk of overordering. But at the moment, there’s no formal system in place for that kind of collaboration.

**Interviewer:** And are there any resources or support systems available to assist community pharmacies in improving their management of returned medicines and wastage?

**Participant:** There are some resources available, like guidance documents from the Health and Social Care Board, but I find them quite dense and difficult to navigate. I think more practical support, like training sessions or toolkits, would be really helpful.

**Interviewer:** And can you share any innovative strategies or initiatives your pharmacy has implemented to enhance the management of returned medicines or reduce medicine wastage?

**Participant:** We have a medicine return box where patients can drop off unused medications anonymously. It’s been quite successful in encouraging returns. I’d love to see more innovative approaches, like digital tools to track medication usage or partnerships with local councils to improve disposal options.

**Interviewer:** And what do you envision as a potential future development or improvement in this area within pharmacy practice?

**Participant:** I think there’s huge potential for better integration between pharmacies, GPs, and other healthcare providers. For example, a shared digital system could help us track medication usage and identify wastage hotspots.

**Interviewer:** Is there anything else you’d like to add or discuss regarding the management of returned medicines, wastage, and disposal in Northern Ireland community pharmacies?

**Participant:** Just that it’s a complex issue that requires a coordinated approach. At the moment, it feels like we’re all working in isolation, which isn’t helping anyone. I’d love to see more collaboration and support, both from regulators and other healthcare providers.

**Interviewer:** Lovely. Thank you so much for your insights and participation in this interview. Would you be willing to provide further input or clarification if needed in the future?

**Participant:** Absolutely, I’d be happy to help. It’s an important issue, and I’m always keen to share my perspective.

**Interviewer:** Lovely. Thank you so much for your time.
